# Fit theory: A cancer experience grounded theory emerging from semi-structured interviews with cancer patients and informal caregivers in Manitoba Canada during the COVID-19 pandemic

**DOI:** 10.1371/journal.pone.0269285

**Published:** 2022-07-22

**Authors:** Maclean Thiessen, Andrea Soriano, Jason Park, Kathleen Decker

**Affiliations:** 1 Department of Internal Medicine, Section of Haematology/Oncology, Rady Faculty of Health Sciences, University of Manitoba, Winnipeg, Manitoba, Canada; 2 Department of Medical Oncology and Hematology, CancerCare Manitoba, Winnipeg, Manitoba, Canada; 3 Max Rady College of Medicine, Rady Faculty of Health Sciences, University of Manitoba, Winnipeg, Manitoba, Canada; 4 Department of Surgery, University of British Columbia, Vancouver, BC, Canada; 5 CancerCare Manitoba Research Institute, CancerCare Manitoba, Winnipeg, Manitoba, Canada; 6 Department of Community Health Sciences, Rady Faculty of Health Sciences, University of Manitoba, Winnipeg, Manitoba, Canada; 7 Department of Epidemiology and Cancer Registry, CancerCare Manitoba, Winnipeg, Manitoba, Canada; Yamaguchi University: Yamaguchi Daigaku, JAPAN

## Abstract

**Background:**

It is not clear how changes to healthcare delivery related to the COVID-19 pandemic, including virtual care and social distancing restrictions, have impacted the experience of living with cancer. This study aimed to discover a theory capable of describing the cancer experience, how the pandemic impacted it, and for guiding predictions about how to improve it.

**Methods:**

Between October 2020 and July 2021 digitally recorded semi-structured one-on-one interviews were conducted virtually with adult cancer patients and informal caregivers in Manitoba, Canada. Transcriptions and field notes from the interviews were analyzed using classic grounded theory.

**Results:**

Interviews with 33 patients and 6 informal caregivers were conducted. Fit emerged as the core concept of the theory and describes the relationship between the healthcare system and the unique combination of characteristics each patient has. Good fit results in a positive experience and poor fit in a negative experience. Virtual care improves fit in clinical situations where non-verbal communication and physical examination are not important. Support from informal caregivers improves fit. Social distancing restrictions reduce the ability of informal caregivers to provide support.

**Conclusions:**

The impact of fit on the cancer experience suggests that care delivery should be tailored to both the individual needs of the patient and the intention of the clinical interaction. Developing evidence-based strategies to inform the integration of virtual care into oncology practice, with aim of promoting good fit between patients and healthcare services, is an important future direction.

## Introduction

In March of 2020 in the province of Manitoba, Canada, public health measures and changes to healthcare delivery were rapidly implemented in response to the COVID-19 pandemic. Table 1 highlights many of the major changes implemented in the province during that month, demonstrating both the rapidity and extent of the transformation of public life and healthcare delivery [1]. Regarding cancer care, changes were in-line with those recommended by recognized guidelines [2], and included increased use of virtual care provision by oncology physicians (i.e., telephone visits and the use of videoconferencing through the Microsoft Teams app) and restrictions to informal caregiver (i.e., friends/family supporting a patient) accompaniment to in-person healthcare visits [3, 4]. The many changes were certain to impact the experience of living with cancer. But it was not clear at the pandemic’s onset how best to assess, describe, or understand their impact in a way that could inform future healthcare delivery and research.

**Table 1:** Summary of COVID-19 related bulletins and communications from Shared Health^†^ highlighting rapid transformation of public life and health services delivery in Manitoba, Canada up to March 31, 2020.

Date	Bulletin #	Summary
January 31 2020	1	World Health Organization declares novel coronavirus a global emergence.
February 20 2020	4	Current risk of COVID-19 to Manitoban’s described as "low".
March 12 2020	8	First person with confirmed positive COVID-19 test in Manitoba.
March 18 2020	12, 17	Suspension of elementary school classes and child care from licensed centers to reduce the spread of COVID-19 in Manitoba.
March 18 2020	13	Recommendations to cancel non-essential international travel and self-isolate for 14 days upon return to Canada.
March 20 2020	22	Visitor access to all Manitoba hospitals is suspended, except for compassionate reasons considered on a case-by-case basis.
March 20 2020	23	Suspensions/delays to elective and non-urgent surgeries.
March 20 2020	n/a	Province wide state of emergency declared by Premier Brian Pallister.
March 27 2021	33	First COVID-19 reported death in Manitoba reported.
March 31 2021	37	103 total number of lab confirmed cases in Manitoba.

† Shared Health is the organization responsible for planning, co-ordination, and administration of healthcare services in the province. See: https://sharedhealthmb.ca/news/archive/2020-news/

It was hypothesized at the start of the pandemic that the patient experience would be negatively impacted in two main ways. Due to the complex multi-disciplinary nature of cancer care, care co-ordination is considered to be an important marker of quality care [5]. Poor care co-ordination has been linked to increased supportive care needs and poorer psychosocial outcomes [6]. Due to the complexity of oncology care, and the fact that changes to healthcare delivery evolved rapidly, it was hypothesized that cancer patients in Manitoba may experience a decrease in how well their care was coordinated. This hypothesis is supported by a report of oncologic epidemiologic and health service delivery data from Manitoba captured during the pandemic and compared to pre-pandemic provincial historical trends (January 1 2015 –March 15 2020) using an interrupted time series approach [4]. This report identified that in April 2020 Manitoba experienced a 20% decrease in new cancer diagnosis (ratio = 0.77, 95% CI 0.67–0.87) and a 43% decrease in surgical resections (ratio 0.57, 95% CI 0.45–0.70). The identified decreased rates of malignancy diagnosis and resection likely reflect the abrupt shift that occurred in terms of care delivery in the province, including reductions in screening programs, limitations in OR time and decreased availability of in-person care. While these changes were evolving, it was easy for the researchers to imagine the experience of patients and their loved ones, perhaps concerned about new symptoms suspicious for cancer and unclear about how to safely seek appropriate medical care, or for those waiting for, in some cases life-saving, surgical procedures unsure when and if these would be occurring and who would be contacting them to inform of them of next steps. Importantly, these metrics quickly normalized, with no statistically significant differences from previous years being identified in terms of cancer diagnosis and surgical resections by August (ratio = 0.96, 95% CI 0.87–1.04) and July (ratio = 0.92, 95% CI 0.83–1.01) 2020, respectively [4]. But despite the return of these important metrics to pre-pandemic times, cancer care service delivery in the province continues to be in a dynamic state at the time of this report. Oncology clinicians and administrators in the province continue to react to threats such as new COVID19 variants while at the same time embracing new knowledge and opportunities to better care for cancer patients through innovations such as COVID-19 vaccines and antibody treatment. The relatively dynamic state of service delivery, especially at the onset of the pandemic when this study was planned and then continuing on in the following months and years as the pandemic continued to evolve, support the hypothesis that the quality of care coordination was likely impacted in a way that negatively impacted the experience of receiving it.

Additionally, the routine use of telephone visits between clinicians and patients was suspected to have a negative impact on the experience of receiving care. The use of telemedicine for clinician-patient communication has been demonstrated to be safe and acceptable to patients in settings including psycho-social and genetic counselling, and post-treatment surveillance [7]. However, it has also been demonstrated that telemedicine, including telephone visits, may be problematic for some patients–including the elderly [8]—and in contexts where “bad news” is being shared [9]. The provision of care through virtual care, including telemedicine dramatically increased in the province at the onset of the pandemic. In April 2020, while the number of visits did not decrease compared to pre-pandemic trends (*p* value = 0.868, ratio = 0.99, 95% CI 0.91–1.08), the number of in person visits did–declining by 52% as care was transitioned to virtual methods (*p* value < .001, ratio = 0.48, 95% CI 0.38–0.58) [4]. Importantly, unlike rates of diagnosis and surgical resection, the rates of virtual care delivery have not returned to those comparable with pre-pandemic trends [4]. While data are not available stratifying the rates of virtual care between demographic categories such as age or purpose of visit (i.e., for sharing of bad news), it is highly probable that provision of care through a virtual format was not optimal in some instances, supporting the hypothesis that virtual care likely had a negative impact on the patient experience.

Theory plays an important role in scientific discovery [10–12]. Theory based approaches utilize theories, frameworks, and models [13, 14] to guide scientific exploration and can help researchers identify their research questions, determine the appropriate methodological approach, and interpret their findings [15]. They facilitate characterization of key concepts and their relationships in a systematic way, resulting in research findings that build on pre-existing knowledge of the phenomena understudy [14]. Theory-based approaches are recommended as part of implementation and evaluation strategies by groups such as the Medical Research Council and the Canadian Institute of Health Research [14, 16, 17]. However, an appropriate theory-based approach could not be identified to guide scientific exploration of how the pandemic impacted the experience of living with cancer. This was due to the many simultaneous changes occurring in the province both to healthcare delivery and public life, making the identification of an appropriate theoretical framework to guide exploration challenging. For instance, the Ambulatory Oncology Patient Satisfaction Survey (AOPSS) is commonly used by many Canadian oncology centers to assess the patient experience [18] and is identified as a tool for assessing patient satisfaction in the Cancer Experience Measurement Framework [18]. It evaluates many domains of the patient experience, including the emotional support provided by healthcare providers and their ability to alleviate anxiety and stress. If the AOPSS was deployed to evaluate the ambulatory patient experience, and it was concluded that healthcare providers ability to alleviate anxiety and stress was worsened from pre-COVID baseline measurement, one would be left wondering what this finding really represented given the turmoil associated with the pandemic. Possible explanations might include: increased baseline of stress and anxiety in the patient population due to rapid changes in healthcare delivery [19]; virtual care negatively impacting the ability of healthcare providers to support patients’ emotional needs; or the pandemic negatively affecting healthcare providers ability to provide empathic care [20, 21]. The relative uncertainty regarding what to evaluate and how to interpret findings suggested that a holistic and exploratory approach for characterizing the impact of the pandemic on the cancer experience would be of value.

Classic grounded theory was first described by Barney Glaser and Anslem Strauss in 1967 as a method for discovering theory through rigorous data analysis [22, 23]. Originally developed in the field of sociology, it has become widely used in many health sciences disciplines including nursing, social work, and medicine [24]. Two hallmarks of classic grounded theory make it the ideal approach for developing a theoretical understanding of the cancer experience during the pandemic: constant comparison and emergence. Constant comparison involves iteratively collection and analysis of contrasting data to identify and characterize common concepts and how they relate [22, 23]. For instance, data reflecting the lived experiences of patients receiving cancer care before and during the pandemic is valuable for understanding the experience of living with cancer before and during the pandemic, how changes to health services delivery impacted the cancer experience. Emergence identifies that the course of exploration is guided by what is learned as the study evolves. By allowing the course of exploration to emerge as the study evolves, and not relying on pre-conceived assumptions or frameworks, the resulting theory emerges from the data [22, 23, 25]. A methodological approach that includes emergence is especially valuable in the context of rapidly changing care contexts such as the pandemic, as it could not be known a priori what was happening in the lives of cancer patients, their friends and families, or what changes the next wave might bring.

This study was undertaken to develop a theoretical understanding of the pandemic on the cancer experience in Manitoba, Canada. The key aims were to discover a theory useful for: 1) describing the cancer experience and how to improve it; 2) understanding how the COVID-19 pandemic impacted the cancer experience; and 3) addressing the guiding research questions. The guiding research questions were selected based on the a priori assumptions described above regarding how the changes to healthcare delivery might impact the cancer experience, recognizing that what emerged *in the field* during data collection and analysis would lead to the identification of additional directions for exploration [22, 25]. The initial guiding research questions were:

1. How have the changes to health services delivery implemented due to the COVID-19 pandemic impacted the experience of living with cancer, including coordination of care and information provision by healthcare providers?
2. How can the provision of virtual cancer care be optimized?

## Materials and methods

Institutional and ethics board approvals were obtained from the Research Resource Impact Committee at CancerCare Manitoba (2020–14) and the Health Research Ethics Board (HS23979; H2020:264) at the University of Manitoba prior to initiation of study activities. Potential participants were selected from respondents to a co-occurring electronic survey being conducted as part of the effort to evaluate the impact of the COVID-19 pandemic on the patient experience in Manitoba, Canada. In brief, the survey used validated questionnaires to evaluate the impact of the pandemic on patient’s perceived coordination of care [26] and satisfaction with cancer-related care [27]. Additionally, demographics and questions regarding satisfaction with telephone and video-conference appointments with clinicians were included. Survey respondents were cancer patients, age 18+, receiving IV or radiotherapy treatments. They were recruited using posters, brochures, and advertisements integrated into appointment reminders. The results from the survey have not been reported to date.

Participants for this study were selected from the group of survey respondents who, as part of their survey responses, consented to be contacted for research opportunities. Survey responses were used to guide initial purposive and then theoretical sampling. Initially, this involved selecting participants for interviews in a way that maximized diversity in terms of demographics, cancer type, treatment intent, and ratings of satisfaction. As the study progressed and the theoretical concepts began to emerge, participants were selected in an effort to explore and develop specific concepts of the emerging theory. As an example, towards the end of the study some individuals being treated with non-curative intent were selected to explore how the meaning and value of time had been impacted by the pandemic. This concept had emerged in earlier interviews and had quickly become well characterized as it related to the curative intent setting. Participants were not known to the researcher prior to the study, except in one instance where a respondent was a patient in the researcher’s medical oncology practice. Participants were informed of the researchers’ professional credentials as part of the process of obtaining informed consent for participating in this study. Participants for this study were approached via the telephone and/or email address they provided through the survey.

As the study progressed the inclusion criteria were expanded to include informal caregivers. This decision was made primarily for reasons motivated by theoretical sampling and saturation. Prior to formally recruiting informal caregivers, it was not uncommon for the participants recruited as “patients” to identify that, in addition to being cancer patients, they simultaneously were informal caregivers for parents or spouses living with cancer. In the data they shared during the interviews, it was clear that their roles as informal caregivers impacted their experience as patients in different ways prior to and during the pandemic. However, it was not always clear which aspects of their experience were related to their roles as “patients” or “informal caregivers”. Therefore, in order to more fully understand the experience of living with cancer, and the impact of the COVID19 pandemic on it, the decision to include non-patient informal caregivers. This facilitated comparing and contrasting data collected from those recruited as “informal caregivers” that may or may not be living with a personal diagnosis of cancer with those recruited as “patients” in order to reach theoretical saturation in the concepts that emerged. Snowball sampling was used to recruit informal caregivers. Theoretical saturation of the concepts relevant to the informal caregiver interviews was reached rapidly, with only six informal caregivers needing to be interviewed. This was likely because the concepts that were explored had already been partially defined through the interviews with the patient participants that had shared relevant data from their experience as informal caregivers.

After informed consent was obtained, data collection was conducted through semi-structured interviews conducted over the telephone or via Microsoft Teams. An interview guide was developed by the authors prior to initiation of the study (S1 File) and was used initially, but revised as the study progressed to provide structure for iterative exploration of the concepts and themes that emerged. The interview guide was not formally pilot-tested as it was expected to change in response to the findings of data analysis as the study progressed [22, 23, 25]. The interviews were conducted one-on-one with the participants, except in one case where an informal caregiver was also present and contributed responses to interview questions. Digital audio recordings of the interviews and field notes were made. During one interview, the digital audio recorder malfunctioned, and a recording of the interview was not made; however, the fieldnotes from the interview were retained. The digital audio recordings were professionally transcribed and de-identified. Participants did not receive copies of their transcripts for comment or correction. NVIVO 12 (QSR) software was used to digitally store and organize collected data and for manual coding, no automated coding features were used.

Data analysis was conducted using classic grounded theory as outlined by Glaser [22, 25] and Holton [23]. Data analysis began following completion of the first interview and continued until theoretical saturation occurred. Constant comparison and theoretical sampling were used throughout the study. Memoing was utilized throughout data analysis. The coding process progressed through open, selective and theoretical coding in keeping with methods well described elsewhere [22, 23, 25]. Data collection, analysis, and drafting of the manuscript was conducted by the first author (MT), a researcher and practicing medical oncologist who has conducted and published previous work employing classic grounded theory methods [28, 29]. He has received training in conducting classic grounded theory research as well as qualitative research methods in general at the masters and doctoral level. Rigour was addressed using the eight practices for enhancing rigour in grounded theory research as outlined by Chiovitti et al. [30], which are summarized in Table 2.

**Table 2:** Methods of rigour employed, adapted from Chiovitti et al. (2003).

Standards of Rigour	Suggested Methods of Research Practice	Application of Rigour
**Credibility**	Let the participants guide the inquiry process	Initial guiding research questions were used to inform the semi-structured interview guide for the initial interviews. As the study progressed, it was identified that the guiding research questions only addressed a small portion of what was discovered to be really "going on" [22, p. 97] for the participants and the interview guide was modified iteratively to facilitate exploration of emerging themes.
	Check the theoretical construction generated against participants’ meaning of the phenomenon	This was done through the course of the interviews as the concepts emerged, participants were asked questions designed to explore the emerging concepts further and invited to reflect on the researchers understanding of the concepts, and how they contrasted with their own experience.
	Use participants’ actual words in the theory	Both long and short quotes are used throughout the results section of the manuscript.
	Articulate the researcher’s personal views and insights	The researcher (MT) is a practicing medical oncology clinician, whose research interests are in supporting the aspects of an individual’s life not directly related to seeking and receiving healthcare during the cancer journey. The researcher believes that high quality is that which attends to the biomedical pathophysiology that a "patient" is diagnosed with and also supports them as a person, striving to respect their relationships, commitments, and goals for the future.
**Auditability**	Specify the criteria built into the researcher’s thinking	Relevant to this work, the primary researcher’s (MT) work regarding the experience of living with cancer is informed by Freire’s critical education theory [31], and the identity theory of Stets and Burke [32]. While not intended to directly build on the researcher’s previous work, the two previous grounded theories authored by the researcher [28, 29] articulate the researchers a priori assumptions—such as those regarding the impact a cancer diagnosis has on identity and information quality impacts the cancer journey.
	Specify how the participants in the study were selected	Participants were selected in several rounds based on their survey responses and demographics. Initially, participants were selected in a way that was likely to result in diverse data and responses, guided by age, gender, type of diagnosis, treatment intent, and responses to the survey questions. As the study progressed, groups of participants were selected in a way that was predicted by ongoing data analysis to facilitate careful comparing and contrasting of data regarding specific concepts. For instance, respondents being treated with curative and non-curative intent were specifically chosen in one of the selection rounds in order to compare their responses regarding a number of emerging concepts including how the pandemic impacted the non-patient aspects of their lives.
**Fittingness**	Delineate the scope of the research in terms of the sample, setting and the level of the theory generated	The selection sample is described in the methods and results section (see Table 3). The level of theory (i.e., middle-range theory) is addressed in the opening paragraph of the discussion section.
	Describe how the literature relates to each category which emerged in the theory	The findings related to the guiding research questions are discussed in relation to the current literature, and the framework fit theory provides. A complete discussion of how each category that emerged relates to the literature is outside of the scope of this report.

## Results

Thirty-nine participant interviews (33 patient and 6 informal caregivers) were conducted between October 2020 and July 2021. Demographic data and interview characteristics are presented in Table 3. The following sections describe the theory that emerged including key concepts, their relationships, and the impact of the COVID-19 pandemic on them. The motivating research questions are addressed in the discussion section.

**Table 3:** Participant and interview characteristics.

Characteristic	n	%	Average (min—max)
**Patient Participants** (n = 33)			
Female	18	55	
Male	15	45	
Age at Date of Interview†			62 (40–82)
**Cancer Type**			
Breast	10	30	
Lymphoma	6	18	
Prostate	3	9	
Colorectal	3	9	
Multiple Myeloma	3	9	
Other ††	8	24	
**Reported Treatment Intent**			
Curative	13	39	
Non-Curative	19	58	
Not Sure	1	3	
**Informal Caregiver Participants** (n = 6)†††			
Female	4	67	
Male	2	33	
Age at Date of Interview			61 (46–76)
**Relationship**			
Spouse	5	83	
Sibling	1	17	
**Cancer Type**			
Breast	1	17	
Lymphoma	1	17	
Prostate	1	17	
Colorectal	1	17	
Other	2	33	
**Reported Treatment Intent**			
Curative	3	50	
Non-Curative	2	33	
Not Sure	1	17	
**Interview Characteristics**			
Total Number of Interviews	39		
Length of Interview (minutes)			45 (18–120)
Number of Telephone Interviews	32	84	
Video Conference Interviews	6	16	

† One patient participant did not provide a date of birth.

†† Includes 1 case each of endometrial, gastric, hepatic, lung, melanoma, sarcoma, and tonsillar malignancies.

††† Of note, many patient participants shared their experiences acting as informal caregivers for others, including friends and family members who had been diagnosed with cancer, and this data was included in the study as it was considered relevant.

### Fit theory

**Fig 1:**
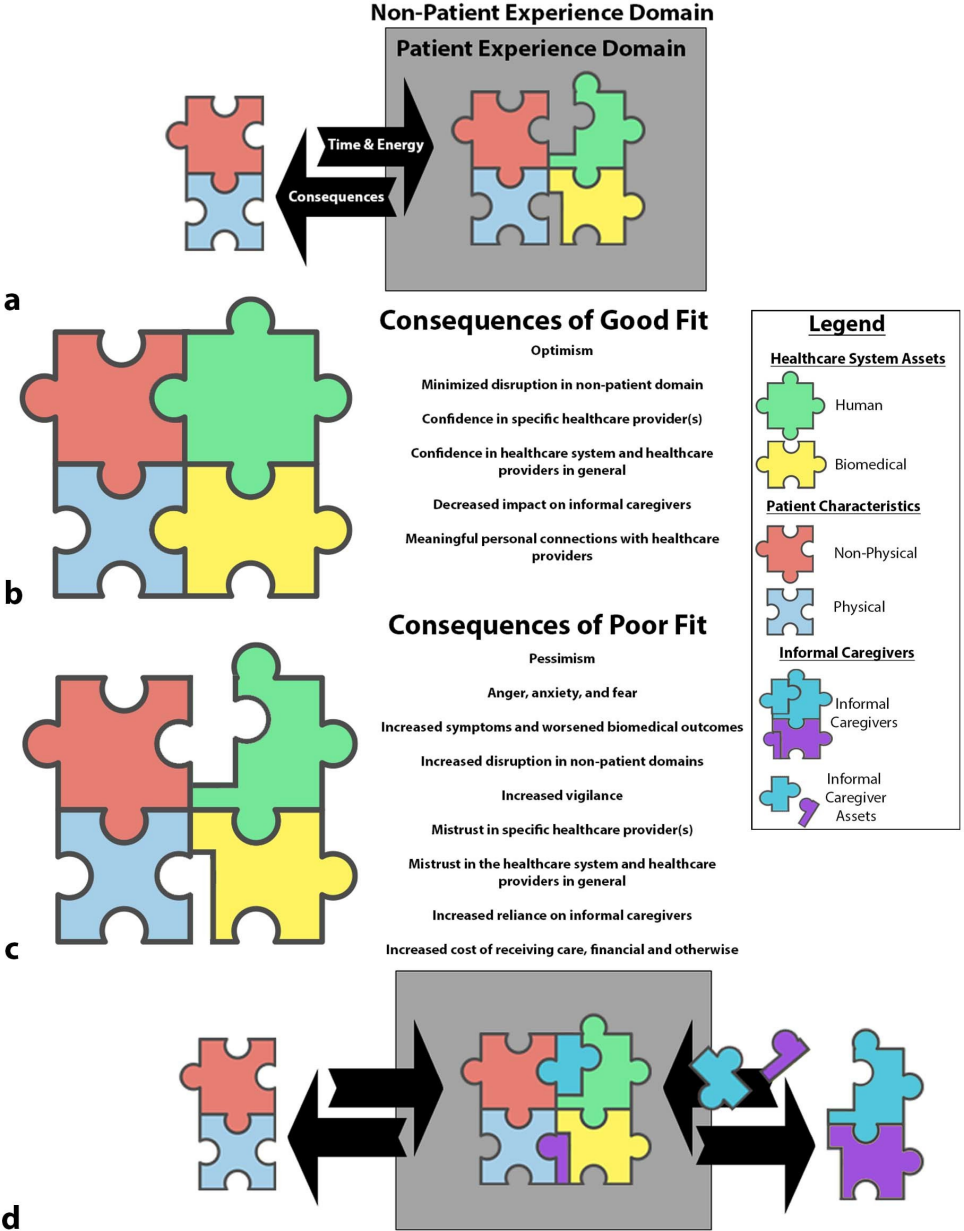
Simplified illustrative model of fit theory. (A) The cancer experience can be described as occurring in two domains: the patient experience domain and the non-patient experience domain. Participation in the patient experience domain requires an investment of time and energy from the patient and is associated with consequences. (B) Good fit between patient characteristics and healthcare assets results in an improved experience of both receiving care, and in the other aspects of the patient’s life outside of their role as a cancer patient. (C) Conversely, poor fit, impacts both the patient and non-patient experience and results in increased needs from informal caregivers. (D) Informal caregivers provide assets that improve fit between the patient and the healthcare system.

For both patients and informal caregivers, the experience of living with cancer can be viewed as divided between two domains: the patient experience domain and the non-patient experience domain (Fig 1A). Fit, the core concept of this theory, refers to the quality of fit between the patient’s characteristics and the assets of the healthcare system—an interaction occurring in the patient experience domain. A better fit between healthcare assets and the characteristics of the patient results in a superior experience for the patient and their informal caregivers (Fig 1B). Conversely, when poor fit occurs, it negatively impacts the cancer experience (Fig 1C). In addition to the healthcare system, informal caregivers provide assets to support the patient, resulting in better fit between the patient and the healthcare system (Fig 1D).

The patient experience domain involves healthcare assets, the characteristics of the patient, and the interface between them. Healthcare assets are the aspects of care that the healthcare system provides to patients. These are divided into two types: 1) biomedical assets and 2) human assets. Biomedical assets are the resources related to the physical delivery of biomedical care, including the personnel and technical resources necessary to obtain accurate diagnosis and administer treatment. Human assets are the tools and resources necessary for providing care beyond simply obtaining an appropriate diagnosis and administering biomedical treatments. Human assets include those that result in patients and their friends and family identifying that they have been treated with respect, dignity, and in a way that engenders trust in their healthcare providers and the healthcare system.

The non-patient experience domain includes the aspects of the individual’s life not directly related to receiving healthcare. This domain includes roles, relationships, and activities such as being a spouse, working, raising children, or involvement in a hobby. In most cases, these roles and relationships originate prior to the diagnosis of cancer.

The characteristics of the patient are divided into 1) physical characteristics, and 2) non-physical characteristics. Physical characteristics are those that interact directly with the biomedical assets of healthcare system. Examples include the geographic location of the patients and the specific biology of the cancer being treated. Non-physical characteristics include the individual’s mental and social needs, strengths, beliefs, and values. Examples of non-physical characteristics include, but are not limited to, having a spiritual faith system that is a source of hope, or having a fear of needles.

#### The connection between the patient/non-patient experience domains and the pandemic

The motivation to engage as a cancer patient with the healthcare system emerged as motivated by the preservation of the roles and relationships in the non-patient domain. The benefit of receiving treatment in the patient domain was described in terms of the years or months expected to be added onto one’s lifespan, or the chance of cure. This was commonly linked by participants to the desire to continue to participate in the activities that gave their lives meaning. Occasionally, engagement with the healthcare system was related to external pressure from friends or family:

… even at the beginning, when I got that diagnosis I had already decided not to take treatment. […] So, then my [spouse] really, really wanted me to take the treatment. So I said, OK, I’ll take it for whatever. (Participant #23)

How fit impacts the aspects of the patient’s life occurring in the non-patient experience domain is variable and emerged as related to the number and flexibility of roles in the non-patient domain. Poor fit was identified to result in a higher level of disruption in the non-patient domains of those living with dependents and/or actively involved in the workforce pre-diagnosis. For instance, one retired patient with no dependents described the impact on their life following a cancer diagnosis as restricted to limiting their ability to travel and participate in hobbies, which was not described as a major loss (Participant #24). In contrast, a younger patient whose employer had allowed them to work from home while receiving treatment described their loss of connection with their workplace:

You feel like you’re really missing out because you’re at home and they’re all having lunch, and they’re–I don’t know, you just feel like you’re missing out on things. You’re not important enough, you’re not doing your job, you’re not doing your part. […] I know I was doing a lot from home, yet it doesn’t seem like it when you’re not there in person. (Participant #21)

For patients experiencing cancers in the curative context, the COVID-19 pandemic was identified as resulting in decreased disruption in the non-patient experience domain of their lives. Participants identified that the pandemic had stopped, or slowed down, many of the social and work activities that would have otherwise been going on. In a sense, there was less that they were missing out on while receiving treatment than if they would have been diagnosed and treated for cancer during non-pandemic times:

Having the pandemic at the same time, maybe reduces the overall impact […]–if the pandemic had come separate from my cancer treatment, then that would have been two years shot. (Participant #27)

For those being treated with non-curative intent, the pandemic resulted in a loss of opportunity for participating in meaningful activities and events like travelling and spending time with friends and family:

[As] the disease progresses and I get ill or more ill, I can’t get back that time. So instead of, you know, enjoying things with–I have three grandkids and like most grandparents that’s a highlight of my life. I really have struggled with the fact that I can’t spend as much time as I’d like to with them. And the same with my own children and their partners. So, I actually am somewhat bitter about COVID for that reason. (Participant #10)

The experience in the non-patient domain for informal caregivers supporting those living with incurable cancers was also impacted by the pandemic. Many of the informal caregivers identified loss regarding inability to travel and engage in social activities with the patient they were supporting. One informal caregiver described the experience of not being able to have their family together due to public health social distancing measures:

I can tell you that there’s been a lot of frustration with something as simple as Christmas day. So, those times have been stripped away from this family–I can only speak about our family–where you were not allowed to, you know, attend somebody’s home. […] Birthdays have been stripped of that nature, as well. And those are really, really important milestones for a [parent dying of cancer], and for me watching it, it’s really not acceptable. (Participant #36)

### Types of fit

Three types of fit emerged through data analysis including fit between: 1) physical characteristics and biomedical assets; 2) non-physical characteristics and human assets; and 3) non-physical characteristics and biomedical assets. Table 4 summarizes these three types, their respective sub-types, and the impact of the pandemic on each.

**Table 4:** Summary of type of fit taxonomy and impact of the pandemic.

Patient Characteristics	Healthcare System Assets	Sub-Type	Description	Pandemic and Fit	Description of Pandemic Impact
**Physical**	**Biomedical Assets**	**Location of Biomedical Assets**	*Describes how the physical journey to care impacts the cancer experience*.	+	Virtual care results in more convenient access to clinicians.
*The type of fit describes the relationship between biomedical assets*, *including access to diagnostic and treatment services*, *and the physical properties of the patient (e*.*g*., *geographic location*, *the features of their malignancy*, *and anatomy required for venous access)*		**Diagnosis and Disease**	*Describes the experience of traversing through diagnostic process and procedures to reach an accurate medical diagnosis for a newly identified symptom or finding*.	-	Virtual care was identified as a barrier to accurate diagnosis in some situations.
		**Treatment and Disease**	*Describes the experience resulting from how disease responds to treatment*.	= /-	With few exceptions, participants did not identify that the effectiveness of their anti-cancer treatment had been adversely affected by the pandemic.
**Non-Physical**	**Human Assets**	No discrete sub-types emerged from data analysis		-	Virtual care limits non-verbal communication between care providers and patients which is important for building trust and supporting patients in getting their information needs met. Social distancing restrictions limited informal caregiver access to in person healthcare visits, resulting in a decreased capacity for them to enhance fit.
*This type of fit describes the relationship between the non-physical characteristics of the patient and non-physical health services assets (e*.*g*., *the attitudes*, *personalities*, *and mannerisms of care providers*.					
**Non-Physical**	**Biomedical Assets**	**Treatments and Procedures**	*Describes the experience of receiving treatments*, *including the physical procedures related to receiving it and the experience of living with side-effects and toxicities*.	-	
*This type of fit describes how non-physical patient characteristics impact the experience of receiving biomedical treatment*. *A key point of contrast is that*, *unlike fit between physical and biomedical assets*, *this type of fit is characterized by the patient’s expectations of what will happen*.		**Healthcare Facilities**	*Describes the experience of receiving care through various health services facilities*, *not related to the experience of navigating to and from biomedical assets*.	-	

+ Better fit

- Worse fit

= No change

#### Physical characteristics and biomedical assets

Three sub-types of fit between physical characteristics and biomedical assets were identified: 1) location of biomedical assets and the patient, 2) diagnosis and disease, and 3) treatment and disease.

*Location of biomedical assets and the patient*: *Accessing healthcare*. Geographically, the closer biomedical assets are to the patient, the better the fit. For instance, a patient in his 80s described the impact of recent changes to the location of where his treatment was being administered–a change from what can be described as a move from a better to worse fit:

Well right now I’m at [hospital #1] with my treatment and that’s oh five to 10 minutes away from where I live. So, it’s great. But beginning next month they switched me over to [hospital #2]. And that’s about a 45-minute drive [both ways] for us… It’s the winter time, if it was spring, summer or fall it wouldn’t be a problem. [But now] it could be a problem just because of weather. (Participant #3)

The ease of physically navigating facilities housing biomedical assets is also contained in this category of fit. One participant shared the experience of being at an unfamiliar cancer center that was challenging to navigate. They described their experience as they rushed to meet a new clinical team for the first time as follows:

My first experience was, “Oh my goodness, is this the kind of ship they run, it’s leaking, and boy, I want to get off before it sinks.” […] there’s so much anxiety […] they give you a number, they don’t tell you what it means, you either get a P number, an E number, […] but it doesn’t tell you what you do when that number flashes by, and what it means, and even where to go. (Participant #33)

The introduction of virtual care in response to the pandemic was identified as having a mixed effect on fit. While additional ways in which virtual care impacted fit are discussed in the following sections, virtual care was identified to improve access to healthcare providers. In particular, the elimination of the need to travel to healthcare centers to meet with physicians was consistently identified as increasing convenience:

For me it was absolutely fine, it felt very comfortable. And because I live remotely it was nice that I didn’t drive the hour in to kind of go through, going into a hospital and everyone is a little anxious now and we’re all wearing masks. Just to be able to sit in your living room. I happened to be in my car. And I just kind of pulled over and had a conversation. So that was nice, I actually preferred it. (Participant #15)

However, virtual care also introduced technical complexity and additional opportunities for fit to be compromised unexpectedly due to a poor connection. Unfamiliarity with the videoconferencing platform being used by clinicians, poor computer literacy, and poor cellular and wi-fi signals were identified as challenges participants experiences to receiving effective virtual care. One participant identified that the videoconferencing platform used in their virtual appointment was not compatible with their hearing aid, resulting in them not being able to exchange information with the healthcare provider (Participant #1).

*Diagnosis and disease*: *Identifying and characterizing disease*. Throughout the cancer journey, patients described seeking medical attention to obtain a diagnosis following the development of new symptoms or following abnormal screening results. Patients who described a straightforward path from initial presentation to diagnosis, such as when a routine cancer screening mammogram leads to a tissue diagnosis in a matter of weeks, provide examples of good fit between the biomedical assets needed to make a diagnosis and disease. When poor fit occurred, the journey from initial concern to diagnosis could take many months. One patient, who described their journey to diagnosis taking almost a year including multiple rounds of imaging, bloodwork investigations, biopsies, and a major diagnostic surgery, shared the following:

Mentally I kept trying to think of what my next steps or the next possible steps might be because that’s kind of my personality. I’m a planner and that’s a hard thing when everyone is unsure of what kind of cancer you have. So that part is hard for me but I think it’s probably hard for everybody to just know that you’re just not making any plans until there’s some official diagnosis. (Participant #10)

While fit in terms of access to healthcare providers was not identified to be significantly impacted by the pandemic, obtaining prompt and accurate diagnosis for a previously undiagnosed physical condition was. Participants shared a number of examples where virtual care, and the lack of a prompt and appropriate physical examination, was perceived to result in a delay in reaching an accurate diagnosis. This is illustrated in the following quote, where an appropriate physical examination led to a cancer diagnosis but only after an initial delay due in part to a virtual assessment:

… I had a phone conversation with my doctor. … And [the doctor] basically told me all the things that I had already done, [they] told me to keep doing them, and I said, “Well, I’ve already been doing that,” and [they] just sort of–I guess it was protocol for [them] to just say to do that, to not take it seriously […] and I thought, this is crazy, like, if I have cancer, I’ve had it for a while now. So, I called back and tried to get an in-person visit so that they could actually see what was going on. And I was able to get an appointment with somebody else in the clinic, who did an examination within a few days, and sort of immediately sent me [for imaging]. (Participant #38)

However, it was also identified that care provided in person had the potential to result in conditions not being accurately diagnosed–identifying virtual care as just one potential factor leading to delayed or missed diagnosis. One participant described being sent home after presenting to physician’s office feeling unwell, being told “no, no, you’re good to go home, everything’s fine” (Participant #25), only to be admitted to the hospital a few days later with “blood clots” (Participant #25).

*Treatment and disease*: *Response and cure vs*. *progression and relapse*. The simplest expression of a good fit between biomedical treatment and disease was observed to be when symptoms, and their impact on the patient’s function, were ameliorated. However, many patients were asymptomatic from cancer, and good fit was evident only on imaging with response or resolution of radiological evidence of cancer, or by the lack of signs of recurrence. Even in instances where cure had likely occurred, and the malignancy was no longer present, patients experienced a lasting impact on their lives.

For those who had completed treatment with curative intent, evaluating whether good fit between treatment and disease had occurred is complicated as the chance of reoccurrence remains a possibility. Re-occurring evaluations conducted by healthcare providers as part of a surveillance strategy, including history and physical examinations, bloodwork, and imaging at regular intervals, were described by patients and their informal caregivers. These evaluations resulted in a cyclical pattern of growing anxiety as these assessments approached, a sense of relief following an uneventful checkup, and then the return of anxiety as the next evaluation approached. Additionally, some described a lasting sense of unease unrelated to the surveillance cycle:

I don’t know, I get strange pains and stuff and you start getting worried—is it coming back? (Participant #34)

Patients receiving cancer treatments intended to control, not cure the malignancy, described the experience of being in a holding pattern, planning their lives around the next assessments of the disease and subsequent instructions from their oncologists:

… he put me on a three-month break. They did another CT scan and extended that break another four months. […] Well, when I first started the treatment it was to be six to nine months. […] After I did my nine months they extended it another few months. And then after they extend again they said, "Oh, we want you to go for a full year,"… (Participant #23)

For patients experiencing disease progression while on treatment, the impact of poor fit between treatment and disease affected both them and their support networks far beyond the experience of awaiting imaging results:

So, first round of chemo didn’t work, now the stem cell transplant hasn’t been successful. At which point, they decide to do the radiation, and they are convinced that, you know, we’ve got this sucker, and we’ve got everybody on board with us. And low and behold, it hasn’t worked. […] The [doctors are] saying, “Oh, we’re starting to run out of options, this thing is spreading by the minute practically,” and do you know how terrifying that must be for [the patient] who’s thinking, there’s something in here growing and trying to kill me? […] Everybody, you know, when you get a diagnosis like this, there are many things that happen. Your support team, people that love you, they’re so overwhelmed, they’re so–everybody was so rooting for us and was so sure. And somehow, they don’t know what to say, so they don’t say anything. (Participant #33)

It did not emerge from the data that fit between anti-cancer treatment and disease was significantly impacted by the pandemic. Some participants did describe that their elective procedures—such as breast reconstruction post curative surgery—had been delayed. Aside from one patient who believed they experienced a recurrence related to a delay in the receipt of curative radiation treatment due to the pandemic and experienced a relapse of their malignancy (Participant #04), no patients described that their anti-cancer treatment had been altered due to the pandemic in a way that resulted in a worse outcome. One participant who received care both before and during the pandemic stated:

It hasn’t been much different, the type of care that I’m receiving. I go for chemo every second week, so that really hasn’t changed. (Participant #03)

#### Non-physical characteristics and human assets: Respect, trust, and connection

Good fit between the human characteristics of the patient and the human assets of those providing healthcare results in an experience, where, at a minimum, the verbal and non-verbal interactions the patient has with healthcare staff does not negatively impact the experience of receiving healthcare. Healthcare providers that took care to offer extra physical comforts such as simply asking “do you want a blanket?” (Participant #21) for patients waiting for a treatment, paying attention to minor medical issues (Participant #14), or having a physician take the time to answer questions in a relaxed and patient manner, were just a few of the many interactions contributing to good fit.

Being treated with disrespect, dealing with healthcare providers who appeared disorganized, and experiencing deviations from how they expected to be treated had the potential for a lasting negative impact on fit. Negative emotional reactions, such as anxiety, to both individual healthcare providers and in the healthcare system in general where described by participants when these types of events occurred. One participant described a healthcare provider as “a scatterbrain … [who] always seems rushed” (Participant #21). The participant (#21) and their informal caregiver (#21) provided an example of an appointment with the healthcare provider where the objective was to review CT imaging and the doctor did not know the purpose for the meeting. They described themselves as revisiting the negative experiences with this physician in their minds and worrying about their next visit with the them as they went about their lives. This participant contrasted these experiences with those they had with another physician who was part of their multi-disciplinary care. They described the physician as “… unbelievably nice. [They] get a gold a star” (Participant #21). The physicians worked in different buildings, and the participants described increased anxiety entering the building where the physician they described as a “scatterbrain” worked, compared to the one they described as “unbelievably nice”.

The increased use of virtual care was identified to impact both the interactions and relationship between patients and healthcare providers in significant ways. In contrast to improving fit in terms of access to healthcare providers, as previously discussed, the fit between the human characteristics of the patient and the human assets of the biomedical system emerged as negatively impacted. Participants shared that seeing the healthcare provider’s face and observing body language was an essential part building trust and provided information about whether the healthcare provider was being honest and forthcoming:

… when I would attend the appointments with [them], I found that you’re face-to-face with the physician. You’re face-to-face with the nurse. You have a better understanding. You’re able to ask questions. You can see the sincerity because when you’re a people person, you can read people, you know. If they’re telling you that things are going well or not well, you can tell. (Participant #36)

This was particularly important at the beginning of the patient-clinician relationship. As one participant described, once trust in the healthcare provider was established, virtual care had less of an impact on trust:

Yeah. I think the first meeting is probably a good idea to do if you can, to do in person [or] do Telehealth so there’s a video, so you can see what somebody looks like. Because when you have that connection, you feel like you have a personal connection. But then after that I’m personally fine with the phone too. (Participant #02)

Participants also described that the duration between seeing their oncologist in person impacted fit, by decreasing the confidence that everything was going well and that they were being taken care of. This was illustrated by one patient on surveillance following completing treatment who, at the time of the interview in July 2021, had not been seen in person by a physician since the beginning of the pandemic:

The visits have all been virtual since then, like my checkups. So other than bloodwork, like, I haven’t had any, like, scans or anything. […] But like I said, I–I haven’t had an examination and I don’t really know what I’m feeling for, like, [the oncology physician] asked if there’s any lumps and stuff. I mean, I try but I don’t know because I didn’t find anything the first time either. So. It kind of makes me uncomfortable that way that I haven’t had an examination at all since [the pandemic] started. (Participant #34)

In terms of information sharing, participants described telephone communication as being impersonal and one-sided. In general, this was identified as a good fit for appointments with physicians where one-directional delivery of information by the physician—and a poor fit in situations when information sharing through conversational back and forth was important:

Well because you don’t need an examination if somebody’s just going over you know what your blood levels are your blood sugar or that, maybe something that they’re seeing on an x-ray you don’t have to be examined. So that to me can be done over the phone. […] I think [telephone visits are] a better use of everybody’s time but you have to get that comfort level established. (Participant #03)

The use of telephone communication had other impacts on fit between healthcare providers and patients. Many participants perceived more background distractions on the physician’s side during telephone interactions:

It’s like I’m talking to somebody, like, a telemarketer. I can hear all sorts of people in the background. My doctor’s talking to, I don’t know if it’s [their] nurse or […] clerk […] it’s completely disconnected. (Participant #39)

For conversations involving sensitive and complex information, such as discussing new treatment plans, reviewing imaging, or palliative care counselling, in-person visits were generally identified as a better fit than virtual visits for many participants:

And like I said, the hard part was all this news I got was always by telephone. It was never face-to-face with a doctor so you could ask questions. I mean you’re just in shock, right? (Participant #23)

However, this was not always the case as the non-physical characteristics of the patient were observed to impact the fit of receiving sensitive information over the phone. One participant described how their professional background made receiving the cancer diagnosis over the phone a good fit:

You know what, I’m okay with over the phone, because of COVID. You know, if that makes things more expedient, like, faster, I’m okay with the phone. But again, I’ve got the medical background, I understand more than maybe a lay person. […] I appreciated getting the phone call earlier than I thought I would, and then it’s, like, okay, so this is what it is, now the next step is the treatment and to deal with it. (Participant #37)

Additionally, for some patients being able to be supported by loved ones in their own homes, resulted in a better experience receiving difficult news than through in-person, face-to-face conversation with physicians. The following two contrasting quotes illustrate this:

Like I had a really–anxiety attack, and I thought I’m going to be sick, and [the physician] just–[they] left the room, and [the physician] got some assistant to come in like a while later. […] Like [the physician] came in the room and my [spouse] had gone to the washroom, I said, “Can you wait till my [spouse] comes back?” [the physician] said, “No, […] we found something under your armpit.” Like [the physician] didn’t even wait until my [spouse] came back in the room to tell me. (Participant #21)

… it was about getting [genetic] results back. […] So that was on the phone as well. […] And so I said to the [geneticist], “I have you on speaker phone” and my [sibling] was [there] and my [spouse] was [there] and that’s who I wanted to be there. And so, either way there was a lot of tension […] I ended up crying anyway. […] Even if it was bad news, [the geneticist] wouldn’t have been able to do anything about it. (Participant #02)

While social distancing and the introduction of virtual care impacted fit between patients’ non-physical characteristics and the human assets of the healthcare system, there were aspects of that relationship that were essentially unchanged. Human connections with healthcare providers were still possible and were described to occur in ways consistent with the pre-pandemic experience. The interaction that one participant described with the nurses in the chemotherapy treatment area is one example:

Like, on my birthday, they made me a bouquet of–they just grabbed some gloves and some leaves from their plants, and I ended up having chemo on my birthday. So they gave me a gift card. That was super-nice of them, caring. […] The treatment room hasn’t changed, besides them wearing masks, you know what I mean? (Participant #39)

#### Non-physical characteristics and biomedical assets

The fit between the non-physical characteristics of patients and the biomedical assets provided through the healthcare system was also identified as impacting the cancer experience. The two sub-types emerged including: 1) between human characteristics and treatments/procedures, and 2) between human characteristics and healthcare facilities. The expectations of the patient and informal caregiver played a major role in mediating experience in both categories.

*Treatments and procedures*: *The experience of receiving on biomedical treatment*. The experience of receiving treatments and procedures was observed to primarily be mediated by the expectations of the participants. Expectations were observed to be informed by a number of different sources including healthcare providers, public media sources such as T.V., the internet, information from friends and family, and previous personal experiences. Expectations impacted the experience of receiving treatment in two main ways. When participants expected a treatment or procedure to go poorly, they often described a negative experience leading up to the procedure. For instance, one patient described her ongoing anxiety associated with appointments for port-access and care:

… I got a port put in, but still they had trouble accessing it, and sometimes they’d have to [try] four or five times because it turns out it was twisted in there, so I just [suffer] like really, really bad anxiety. (Participant #21)

Additionally, when a treatment/procedure was different than expected or the information provided by healthcare providers did not adequately prepare the patient for what was going to happen, participants described a negative experience:

… and I said, "No, I’ve had enough." I was going for weekly treatments. So–and I was pretty sick all of–the full first year I was on there. Like I–of course–you know what you expect to lose your hair and your nails, and I didn’t expect, like, atrophy in my hands and feet [or] nasal ulcers, [and] body spasms which were really bad. (Participant #23)

In contrast, for some, the experience of treatment is better than expected. One participant shared that the expectations they had developed regarding their own treatment were in part developed from their experience with a family member who had passed away from cancer, and that comparatively, the experience of receiving treatment was a good one:

Oh, I thought I was going to be on the drip all the time and the only drip I get is the calcium once a month. The rest is just the needle in the belly. To me I’m getting away scot-free. (Participant #20)

The impact of the COVID-19 pandemic on the patient experience of receiving treatments and procedures did not emerge as significant. This appeared to be in large part related to the fact that the actual delivery of medical treatments and procedures was relatively unchanged, and a large amount of the support that patients received through one-on-one nursing was still available–both over the phone and in-person during visits to treatment and clinical areas.

*Healthcare facilities*: *Fit between non-physical characteristics and hospitals and clinics*. The physical characteristics of healthcare facilities, including their general condition, emerged as not in and of themselves a major determining factor of fit. The fit between the physical characteristics of the facility and the patient was, in part, related to the other facilities that participants had attended:

But once we saw [Hospital #2], then [Hospital #3], we realized that [Hospital #1] maybe was not quite as great as we thought. […] It was just, you know, it was–to just be blunt, it was a dump. (Participant #32)

However, when describing the care at the facility described as a “dump” the participant highlighted the central role of healthcare providers in creating a positive experience, a factor that outweighed the general condition of the facility:

The nurses and the doctors were very good … the people were great and the attention was great, so. (Participant #32)

Other participants described volunteers and healthcare staff being friendly, and helping them navigate new facilities that had signage that was confusing. It was a consistent finding that positive experiences with healthcare providers were able to overcome perceived physical shortcomings of the facilities themselves. However, instances were not identified where the physical attributes of a facility overcame interactions with healthcare providers described as negative.

While the healthcare facilities did not physically change as a result of the pandemic, an element of uncertainty was added to the task of accessing biomedical facilities in person. For instance, participants described dealing with the sudden presence of long COVID-19 screening lines at healthcare facilities, and being surprised when guidelines that normally restricted informal caregivers accompanying patients in healthcare facilities, were altered:

… the day I checked in, they changed the rules that allowed one visitor. And the expectation was going to be, I’m in there for twenty-one days, no visitors, period. That would have been very, very nasty for my [spouse] and for myself. […] And that probably made a huge difference in terms of morale and attitude and stuff for both of us. (Participant #29)

One important finding was how the pandemic impacted the experience of *waiting* to access biomedical assets–either prior to appointments with clinicians or in treatment areas. Participants described areas where waiting occurred as important places for gathering information and obtaining support from nurses, clerks, and other patients and informal caregivers. Despite social distancing measures, patients described still having meaningful communications with healthcare providers and other patients in waiting areas.

Lastly, the experience of entering biomedical facilities, and receiving services without informal caregivers present was identified for many as a significant challenge. This was especially evident at the beginning of the cancer journey, when facing a novel challenge such as a new treatment, or when meeting a new care team:

… it takes a lot of just fortitude just to stand up and walk through the doors, not having someone beside you. (Participant #02)

#### Informal caregivers and fit

Importantly, across all typologies, informal caregivers emerged as having the potential to improve fit. They were observed to provide both emotional and physical support–enhancing the care that the patient received through the healthcare system. One informal caregiver described the vital role they played in improving fit between the patient and the healthcare system:

… I was working as a middleman between the doctors and nurses, trying to keep abreast of what the results were, what this meant, you know, how were we going to go forward, what problems [patient] was facing. (Participant #26)

Each informal caregiver was identified to possess have a unique combination of assets available to contribute to the care of the patient. A particularly clear example came from one participant who described what each of their three adult children contributed to their care. They identified that one of their three children was a nurse, another was a physiotherapist, and another had a great natural sense of humor (Participant #35). The skills of the nurse were identified as particularly helpful in the immediate post-operative period from cancer surgery and in helping the patient get through chemotherapy. The physiotherapist assisted with rehab and recovery from surgery. The child with the great sense of humor provided emotional support through the cancer journey and helped to lighten the mood.

It emerged from the data that informal caregivers not only improve fit between healthcare assets and the patient, but they also support the patient in the aspects of their lives outside of the patient experience domain. Common examples included helping out with housework or child care. One informal caregiver described the support they provide in addition to assisting with medical care:

… and then through [their] personal life, we support [the patient], [who has] got two small kids. […] sometimes after treatment, you know, if [they’re] experiencing some fatigue, things like that, I like to open up my home and say–because [the patient] wants to spend time with [their] children, [and] just doesn’t want people picking up [their] children to look after them. [They] still want to be there even if [they] need to rest a little bit. So just welcoming [the patient] in our home and having [their] kids play with my kids and just giving her that environment where [the patient] can still really enjoy that, but sort of relax a little bit while, you know, I can make a meal or run after the kids a little bit, things like that. (Participant #36)

Providing support to patients during the cancer journey emerged as requiring the investment of time and energy on the part of informal caregivers–this cost was identified to be proportional in many ways to the fit between healthcare assets and the patient. One informal caregiver described the extent of the emotional support they provided to their partner and how it impacted their ability to contribute to their workplace:

Well cancer changed [patient’s] life; I’ll put it that way. [They were] not an anxious person; [they] didn’t have anxiety at all before cancer. So most of my days are spent making sure [they’re] OK. […] Well I’m lucky–I do have a lot of sick days. (Participant #22)

The impact of the pandemic on informal caregivers’ ability to support patients in accessing services was evident in situations where they were not able to provide in-person support–such as in facilities where strict social distancing measures were in place. This resulted in informal caregivers being unable to support the patient in accessing biomedical assets beyond transporting them to healthcare facilities:

Well obviously I’m worried about [the patient] but I never ever–I made sure that I didn’t add to [their] anxiety before. Everything was positive, like I was as positive as possible till the second [they] walked out the door of the car. (Participant #22)

Additionally, for informal caregivers who did not live in the same household as the patient they were supporting, there were limitations to the support they could provide. For instance, some informal caregivers described not being able to provide help with things like childcare and meal preparation, things they would normally do for the patient on days they were unwell from treatment, due to social distancing restrictions.

However, it was identified that the increased use of virtual care through telephone and video-conferencing improved fit for some informal caregivers. Informal caregivers identified that the convenience of the phone calls was beneficial as there wasn’t a need to provide transportation, which for some required missing work. It was also noted that virtual care supported more informal caregivers being involved in patient visits with physicians, as multiple friends and family members could be connected through conference calls with healthcare providers.

Lastly, disruption to the regular in-person care routine was identified as a cause for concern among many informal caregivers:

And so, when we were told, no, we can’t see [the doctor], that kind of breaks up that, you know, superstition I guess you’d call it. You know? You feel like, well, Jesus, they’re not going to see us and then […] and that brings a lot of worry and tension. (Participant #26)

## Discussion

Fit theory is a middle-range theory that can be used to describe the cancer experience and guide predictions about how the cancer experience is impacted by changes in healthcare delivery. The concepts defined as part of fit theory can be used to guide clinicians and researchers alike in the identification of important relationships and outcomes to consider and explore. For instance, conceptualizing informal caregivers as reducing the consequences of poor fit (Fig 1C) and using the types of fit (Table 4) to identify opportunities to identify opportunities to better support informal caregivers in their roles provides a framework for guiding both clinical practice and research. The grounding of the theory in data collected from individuals with a wide range of ages, a mix of genders, cancer types, and treatment intention, as well as the inclusion of informal caregivers, supports that this theory is applicable for a wide variety of cancer related contexts. These characteristics make fit theory a middle range theory because it is specific enough to “guide research and practice, yet sufficiently general to cross multiple clinical populations and to encompass similar phenomena” [33, p. 11, 34].

Fit theory provides a sensitizing lens with the potential to help inform how clinicians and researchers think about the relationship between healthcare and the cancer experience. For additional guidance on how fit theory may be applied in real world situations see S2 File for worked examples and a template for assisting with its application. To our knowledge, a grounded theory that provides a description of the impact of the relationship between healthcare services, patients, and informal caregivers has not been previously discovered–but this claim is made without a high level of certainty. Given that the work to discover fit theory was performed as a pragmatic response to the evolving COVID-19 pandemic in Manitoba, steps such as performing a rigorous systematic literature review to scope [35] existing cancer experience theories was not performed. This would be a valuable undertaking not only to better identify fit theory’s place in the literature, but also to index which theories of the cancer experience already exist and where further theoretical development is needed.

### Addressing the guiding research questions

The following section will address the guiding research questions using insights gained from through the discovery of fit theory as well as the existing literature.

#### The pandemic and coordination of care

Based on the complexity of providing multi-disciplinary oncology care and the number and rapidity of changes to healthcare delivery occurring in a rapid fashion at the start of the pandemic [3], it was hypothesized that this study would identify issues with how care was coordinated related to the pandemic. Evidence to support this hypothesis did not emerge during this study. By comparing and contrasting the experiences of participants from before and during the pandemic there was little evidence of a meaningful difference in the perception of how care was coordinated. Appointments, tests, and procedures happened as planned and participants did not describe any issues specific to the pandemic in this regard.

However, it is possible that the issues with coordination of care resulting from the pandemic were simply missed and there are two possible reasons for this. First, the semi-structured interviews for this study began in October of 2020 and continued until June of 2021, with most service delivery changes being implemented earlier in the year—closer to the month of March [1, 3, 4]. This timeline resulted in many months passing since the initial, and likely most dramatic, COVID-19 related changes to cancer care, and any data collected in relation to this period may have been affected by recall bias. Ideally, data collection for this study should have begun closer to the March of 2020, but this was not possible as time was needed to secure funding, gain ethics and institutional approvals, and recruit participants. These challenges highlight the importance of serially collecting patient-reported outcome and experience measures on an ongoing basis that reflect the important domains of the patient experience. This would have facilitated a real-time understanding of the impact of the pandemic on the patient experience. Furthermore, this could have facilitated streamlined recruiting for in-depth exploration instead of through the survey that participants used for this study.

The other reason why issues with the experience of care coordination could have been missed is due to how the participant interviews were conducted and the coding structure that emerged. The interviews were structured around questions designed to result in the participants sharing what was important to them regarding their experience. It is possible that a detailed and focused exploration specific to care coordination may have yielded important insights and evidence in support of a significant impact from the pandemic. This, however, would have been at the expense of exploring the issues that emerged as important to the participants (i.e., what was “really going on”). Given our approach, it is reasonable to conclude that any issues with care coordination were either transient or simply overshadowed by the other issues that emerged from the collected data—such as those related to virtual care and the decreased access to informal caregivers in in-person clinical interactions.

#### The pandemic, information provision, and optimizing virtual care

The findings of this study build on what is already known about the provision of oncology care through virtual medicine [7, 36]. Previous work published both before and during the COVID-19 pandemic has identified that virtual care is both safe, and may be preferred by patients in specific circumstances, such as when included as part of surveillance after curative treatment [37–39]. A recent survey study of 108 sarcoma patients during the pandemic in the UK [40], identified higher satisfaction with virtual visits (8.99/10) than with in-person visits (8.35/10). Reduced travel time and expense, and increased convenience were reasons identified for preferring telemedicine care [40]. Through the lens of fit theory, these findings make sense, as they reflect that patients value care that comes with less of an impact on the aspects of their lives occurring in the non-patient domain. The challenge moving forward is to develop strategies to ensure virtual care is conducted in a way that supports both appropriate biomedical care as well as the unique non-biomedical care needs of the individual patient.

Importantly, 48% of respondents in the above-mentioned survey identified that they would not want to hear bad news over the phone, with no differences in preferences identified between age, sex, or education level [40]. Fit theory adds additional insight to this finding as it helps to characterize the important role of personal coping characteristics and informal caregivers in supporting a patient when receiving difficult news. Fit theory suggests that it is likely that some patients would prefer to receive bad news through virtual care so that they could be have the conversation in a physical environment that they are comfortable, such as their own home, with their informal caregivers present, either physically or virtually.

#### Clinical implications

Consistent with previous work, this study identified that informal caregivers add to the care of patients in many ways [41–43], including through assisting with information gathering from doctors and nurses [28]. While it did not emerge directly from the data analysis, Fit theory suggests that it is possible that coordination of care may have been impacted as informal caregivers were not able to attend appointments. Clinicians are encouraged to identify ways to ensure that important information about what to expect from care, including what aspects of care the patient and their informal caregivers are directly responsible for, are communicated to informal caregivers, even if they are not able to attend appointments. For in-person visits, clinicians may consider assisting the patient with recording the visit on their smartphone or other device if a formal consultation recording service is not available so the discussion can be reviewed later with the informal caregivers. This recommendation is supported by previously existing evidence that consultation recording has been demonstrated to positively impact psychological health, well-being, and knowledge transfer [44]. For telephone or virtual visits, including informal caregivers through speakerphone, conference calling, or providing them with link to join video calls are all simple and practical strategies to help patients benefit from the added support of their informal caregivers.

In terms of the provision of virtual care, clinicians need to be aware of the important elements of clinical care that, while present with in-person care, may be at risk with virtual care [7, 9, 36, 40]. In some cases, participants described hearing background noise and experiencing uncertainty regarding whether clinicians were focusing specifically on them. This suggests that like in-person visits conducted in private exam rooms, clinicians should be supported in having appropriate facilities where they can focus on engaging with patients virtually without distractions. Ensuring healthcare providers are provided with dedicated quiet space to conduct virtual care, such as a clinical exam rooms that are adequately equipped to conduct virtual care without risk of interruptions or distractions, will likely improve fit. Additionally, clinicians need to have a low threshold for converting virtual care to in-person visits. Patients at risk of deterioration without seeking medical attention, including the frail elderly, and those without a robust informal caregiver support network, should be assumed to be a poor fit for virtual care alone until proven otherwise.

Based on the experiences shared by participants in this study, developing strategies to inform those receiving virtual care about strategies to optimize clinician visits is important. Coaching patients and informal caregivers to prepare a list of questions to ask clinicians before virtual visits and taking time to develop familiarity with speakerphone and conference call features will likely lead to improved fit. Clinical recommendations regarding virtual care are summarized in Table 5.

**Table 5:** Clinical recommendations* for virtual care.

1. Consider utilizing strategies to ensure informal caregivers have access to information shared during clinical communication. Consultation recording, conference calling, and utilizing speakerphone for in-person visits when informal caregivers cannot be present are recommended approaches when important information is being shared.
2. Dedicated spaces free of noise and distractions should be provided for clinicians to conduct virtual visits.
3. Education for both healthcare professionals and recipients of healthcare regarding optimal practices using virtual care should be provided. Both parties need to be aware of the steps they can take to improve the fit of virtual care.
4. Provide opportunities for patients to opt-in or out of virtual care when medically appropriate. Identifying what will likely be discussed at the visit, and screening for psycho-social distress as well as any new or evolving physical symptoms that would prompt in-person assessment are key aspects of this process likely to improve fit.

* Level IIIC evidence. Expert opinion informed by descriptive research [45].

#### Future directions

Developing educational strategies for patients, informal caregivers, and clinicians around the use of virtual care in oncology, with the aim of improving fit, is an important future direction. Patients and their informal caregivers need to be aware of what to expect in virtual visits, including their limitations, and when to insist on being seen in-person. Given the impact of virtual care on fit, both in terms of biomedical and psychosocial outcomes, this is an area worth exploring through systematic research. Based on the widespread uptake of virtual care during the pandemic in the cancer context, this work would be of utility around the globe.

Additionally, evidence-based strategies are needed to guide clinical decision making regarding when a patient needs to be assessed in-person or when a telephone visit is appropriate. Based on fit theory, such a strategy would include consideration for both healthcare system assets and patient characteristics. For instance, patient preference and geographic location are important from a patient centered perspective in terms of determining whether to provide care virtually. However, time since last in-person clinician visit, the presence or absence of new or evolving symptoms, indication for the visit that is currently being planned (i.e., where the patient is in their care pathway), and characteristics of informal caregivers/support system (i.e., number of people involved, their physical/cognitive ability, and the types of relationships) are important patient characteristics in terms of assessing whether virtual care can be delivered safely. One approach might involve a model involving a hybrid of healthcare provider decisions and patient input. For instance, the healthcare provider role could involve constructing a customized virtual care plan for each patient including whether the patients are eligible for virtual care, including the types of formats they are eligible for (i.e., telephone vs video-conferencing), how often they should be seen in-person, the types of visits where they require in-person assessments, and instructions for under what circumstances the virtual care plan should be revised (including how often it should be reviewed). From the patient perspective, deployment of questionnaires to patients in the days and weeks prior to their appointments using secure electronic methods via smart phone apps or email surveys, facilitating capture of new symptoms, concerns, and, only for eligible patients when appropriate based on their virtual care plan, if they are interested in being assed virtually and what format they would prefer. Important metrics to consider as part of the evaluation and refinement processes, based on fit theory, would include the cost of care delivery, as well as the financial impact on the patient, the amount of time involved in receiving care, satisfaction with care, symptoms, and emotional distress. Fit theory is useful for providing an evidence-based theory as a starting point for constructing a strategy for triaging which patients are appropriate for virtual care and for measuring its effectiveness. However, developing and testing such a strategy will require input from all stakeholders, including patients and their informal caregivers [24] and careful evaluation using an approach suitable for complex interventions [17] and likely involve the use of quantitative and qualitative methods through all phases of development and deployment [46].

#### Limitations

Recruitment for this study was limited to those receiving cancer care in Manitoba, Canada, and not focused on identifying participants with specific cultural backgrounds. Therefore, fit theory should be applied with some caution in other regions and in specific cultural groups. Extending and adapting the theory in other parts of the world and in specific cultural groups is likely a valuable direction for future work. Additionally, recruitment was focused on those receiving IV treatments such as chemotherapy, as well as radiotherapy. While many of these patients received surgery, it is not clear how the cancer experience in instances were only surgery was indicated was impacted by the pandemic.

Lastly, it should be noted that this study was not an evaluation of the quality of the healthcare system or the quality of the cancer experience in Manitoba, Canada before or during the COVID-19 pandemic. Many of the quotes in the results section describe participants having a negative experience with some aspect of their cancer experience, but conclusions about the overall quality of care cannot be drawn from these. The examples were chosen to demonstrate the concepts of the theory, as such those that were the most dramatic, because they were the most illustrative. Standardized approaches, such as using validated patients reported experience measures, including the Ambulatory Oncology Patient Satisfaction Survey, are likely better suited for the purpose of evaluating care [40, 47].

## Conclusions

The experience of living with cancer in Manitoba, Canada was identified as being impacted in a number of ways by the healthcare delivery changes related to the COVID-19 pandemic. Fit theory guides understanding of how the many changes to public life and healthcare delivery impacted the lives of those living with cancer. The findings of this study identify that virtual care, when implemented in a way that provides optimal fit between patients and health services, can improve the experience of living with cancer. However, research is needed to develop evidence-based strategies for integrating virtual care into contemporary oncology practice in way that optimizes fit. Hopefully the findings from this study will assist in cancer care providing good fit for patients and their loved ones, in the new normal and beyond.

## Supporting information

S1 File Initial interview guide used in the semi-structured interviews. (DOCX)

S2 File Applying fit theory: Illustrative case scenarios and application of fit theory. (DOCX)
