# Supplementary material for: Fit theory: A cancer experience grounded theory emerging from semi-structured interviews with cancer patients and informal caregivers in Manitoba Canada during the COVID-19 pandemic
Source: PLoS One. 2022 Jul 22;17(7):e0269285. doi: 10.1371/journal.pone.0269285 (PMC9307189; doi:10.1371/journal.pone.0269285)
Supplement: S1 File — (DOCX) [file pone.0269285.s001.docx]

Initial Interview Guide

Introduction

Thank you for agreeing to participate in this study. This interview should take approximately forty-five minutes and it is being recorded. At any point, you are welcome to take a break or stop the interview all together.

1. Tell me about your experience of receiving cancer care during the COVID-19 pandemic?
2. I understand that you have recently used telemedicine to communicate with your physician due to the recent COVID-19 pandemic. Tell me about your experience using telemedicine?
3. How did you feel about using telemedicine?
   1. What was helpful about using telemedicine?
   2. What was unhelpful about using telemedicine?
4. How would you compare meeting with a physician in person to your telemedicine experience?
   1. Which do you prefer? Why?
5. What do you think should change about telemedicine appointments?
   1. Any advice to physicians on how to make your experience better?
6. What are your thoughts on using telemedicine for future appointments?
7. Is there anything else you would like to tell us about your experience with telemedicine?
8. Are there additional questions you think we should be asking to get a better understanding of patient’s telemedicine experience?

Thank you for taking part in this interview. This concludes your participation in this study. Contact information for myself, my supervisor, and the University of Manitoba Research Ethics Board are listed inside of the consent form if you have any questions, comments or concerns.
