# Supplementary material for: Fit theory: A cancer experience grounded theory emerging from semi-structured interviews with cancer patients and informal caregivers in Manitoba Canada during the COVID-19 pandemic
Source: PLoS One. 2022 Jul 22;17(7):e0269285. doi: 10.1371/journal.pone.0269285 (PMC9307189; doi:10.1371/journal.pone.0269285)
Supplement: S2 File — (DOCX) [file pone.0269285.s002.docx]

**Applying Fit Theory: Illustrative Case Scenarios and Application of Fit Theory**

**Contents**

[Introduction and Outline 2](#_Toc98420846)

[Cases and Analysis 3](#_Toc98420847)

[Case 1: Pat 3](#_Toc98420848)

[Deconstructing Pat’s case 5](#_Toc98420849)

[Identifying Opportunities to Improve Fit 7](#_Toc98420850)

[Case 2: Charlie 9](#_Toc98420851)

[Deconstructing Charlie’s case 11](#_Toc98420852)

[Identifying Opportunities to Improve Fit 12](#_Toc98420853)

[Concluding Notes on Applying Fit Theory 13](#_Toc98420854)

[References 15](#_Toc98420855)

[Appendix A: Fit Theory Analysis Framework Blank Template 17](#_Toc98420856)

[Appendix B: “Pat” Fit Theory Analysis Example 18](#_Toc98420857)

[Appendix C: “Charlie” Fit Theory Analysis Example 19](#_Toc98420858)

# Introduction and Outline

One characteristic of theories generated using classic grounded theory (CGT) is that they can often be used to inform practice (1). While Glaser states that CGT has to be “generated by a trained researcher”(2, p. 17), he goes on to point out that the resulting theory can “be applied by intelligent laymen as well as other professionals”(2, p. 17). This claim is supported by the popularity and numerous of applications of theories generated using CGT. For instance, theories generated using CGT have successfully been used in the research setting to better understand findings of quantitative research; in the education and professional development setting to structure courses and learning activities; and, notably in the case of the grounded theory generated by Brené Brown, to inform self-help tools available to the lay-public (2, 3).

Glaser also notes that, despite CGT theories having numerous real-world application, the topic of application is relatively neglected, and, that in reality, application rarely occurs (4). In response to these observations, as well as to the comments of one of the peer-review of the accompanying manuscript requesting “concrete example[s]” to assist with explaining Fit Theory, this supplement has been developed. It is designed to provide an illustrative example of how Fit Theory can be applied. It includes case examples and analysis to demonstrate how Fit Theory can be used to identify and describe challenges experienced resulting from the fit between the personal characteristics of those living with cancer and health services assets. Additionally, the examples demonstrate how Fit Theory can be used to predict ways that fit, and the resulting cancer journey, could be improved. The case examples presented in this supplement are fictional. They have been developed by combining concepts that emerged from data collected from the participants in the semi-structured interviews in the research study that this is supplement corresponds to, as well as the lead author’s previous CGT studies involving semi-structured interviews with cancer patients and their friends/family (5, 6), and do not represent specific individuals.

The following sections include two case examples. Each case example includes a narrative description of the challenges faced by the individuals, an analysis using Fit Theory, and an example of how Fit Theory can be applied to identify strategies for improving the cancer experience. The analysis and application of Fit Theory is aided by the Fit Theory Template (see Appendix A). Following the two case examples, this supplement concludes with closing comments regarding how Fit Theory, and this supplement, may be applied to inform healthcare delivery as well as enhance healthcare education.

# Cases and Analysis

## Case 1: Pat

Pat is 50 years old and living alone in a rural community one hour away from the nearest medical clinic and three hours from the nearest cancer center. Pat’s partner passed away several years ago of colon cancer. Over the last four months Pat has experienced 15 lbs. of weight loss and increasing right sided chest pain. Pat attributed the weight loss to increased physical activity and the chest pain to muscle strain related to weightlifting at the gym. Pat had been working on getting back into shape to return to the dating scene.

Pat presented to the emergency room (ER) after coughing up a small amount of blood at home a few weeks ago. A chest CT scan was performed and identified a mass in Pat’s right lung as well as a right sided pleural effusion. Prior to discharging Pat from the ER, the ER physician took a sample of the lung fluid for testing, and prescribed Pat some pain medication. The ER physician said that the CT scan looked like lung cancer but that the diagnosis needed to be confirmed. The ER doc said the results from the pleural tap would likely confirm the diagnosis. Pat was told to follow-up with the family doctor for the results of the pleural fluid testing and ongoing management of the chest pain. When the results from the fluid sample were ready a week later Pat’s family doctor called Pat’s cellphone while Pat was driving and told Pat that the fluid confirmed the diagnosis of lung cancer. Pat didn’t have a chance to pull the car over before receiving the news, and just about got in an accident while trying to pull over while fighting back tears after getting the news. The family doctor told Pat that a referral to the cancer center would be made and to wait to hear from the cancer center about an appointment. This was two weeks ago. Pat has an appointment with the family doctor in a few weeks to review pain medications.

Since receiving the diagnosis Pat has been in disbelief. Pat thought that lung cancer was a smoker’s disease. Pat never smoked except for a few cigarettes at parties in high school and occasional marijuana use in college. Pat is also frustrated because no information was provided through the family doctor or the ER physician on what to expect from treatment or if the cancer could be cured. Pat had searched for information on lung cancer after being sent home from the ER and found some information on survival but after reading that the chance of living for 5 years was less than 5% Pat had not searched more online. Pat has a friend whose parent passed away from lung cancer and Pat called the friend to ask questions “for a friend” about lung cancer. The friend told Pat that “lung cancer is a death sentence” but that “it was the chemotherapy not the cancer” that had killed the friend’s parent. The friend also told Pat that immunotherapy would have cured the cancer but that the treating doctor would not prescribe it.

Pat hasn’t been to work since the ER visit. Pat spends the day on the couch waiting for a call from the cancer center with the day and time for the appointments with the cancer specialists. Pat often cries because of the information about prognosis he read on the internet. Pat is afraid of chemotherapy and is hopeful that immunotherapy will be an option. Pat hasn’t shared the diagnosis with friends or family because of being embarrassed about being diagnosed with a “smokers” disease. Pat is ashamed about taking opioid medications and fears becoming an addict.

### Deconstructing Pat’s case

*See Appendix B for the analysis of Pat’s example presented using the Fit Theory Template.*

In terms of personal characteristics, Fit Theory divides these into non-physical characteristics, physical characteristics, and informal caregiver assets. In terms of non-physical characteristics, Pat is living alone after recently losing a partner to cancer. Pat also has beliefs about lung cancer that have led to further isolation from friends and family who may have otherwise been able to provide support. Additionally, Pat has received information that may not be completely accurate from a friend as well as the internet about lung cancer.

In terms of physical characteristics, distance from the cancer center and its associated resources is important, as there may be resources that Pat would access while waiting to be seen at the cancer center if travel was not an issue. The narrative describes that Pat’s pain is under control suggesting that there is a good fit with the pain medication that has been provided. It is not clear how the weight loss and the hemoptysis are impacting Pat’s experience at this moment based on the narrative provided. Pat is relatively young which may positively impact Pat’s ability to tolerate treatment. Since Pat has a pleural effusion, this is considered to be metastatic and incurable disease. However, we do not know anything about the type of lung cancer or the presence of targetable mutations which will have a significant impact on Pat’s treatment course and long-term prognosis.

In terms of informal caregiver assets, Pat has chosen to keep his diagnosis a secret. From the narrative, this appears to be related to Pat’s personal beliefs about the lung cancer diagnosis being a “smokers” disease. In terms of Informal Caregiver Assets, while Pat may very well have family or friends that could provide emotional support and possibly provide insights or assist with access to better informational resources because Pat has chosen to keep the diagnosis a secret and therefore will not benefit from any potential support at this time.

Moving to how Pat’s personal characteristics fit with the available healthcare system assets, it appears there are number of instances of poor fit. In terms of human assets, the narrative describes that little was shared about the lung cancer diagnosis, such as what might have caused it, or what to expect from next steps from the cancer center, additionally Pat was not directed to any additional informational resources as result of the visits with the ER physician or the family doctor. Pat’s informational needs were not met by the healthcare system. This claim is supported by the fact that Pat searched for additional information on the internet and asked a friend question about lung cancer. Given Pat is living in a small rural community, it is possible that few local resources exist to help Pat get orientated about what the diagnosis means or what might happen next. As a result, it can be argued that poor fit exists between Pat’s informational needs (i.e., non-physical characteristic) and the information provided by the individual healthcare providers (i.e., human assets) as well as formal informational resources available (i.e., biomedical assets).

The narrative does not describe much about the way Pat was given the bad news, either by the ER doctor or Pat’s family physician but it appears poor fit existed here as well. The narrative describes Pat being given the news about the pleural fluid results demonstrating cancer cells over the phone, which given the fact that Pat was driving at the time of the call was unexpected in terms of timing. As a result, poor fit can be argued to have occurred between the human assets delivering the news and Pat’s non-physical characteristics, including Pat’s coping skills and responsibilities outside of being a patient (i.e., safely operating a motor vehicle on public roads).

In terms of biomedical assets, Pat’s geographic distance from biomedical care makes Pat’s situation more challenging. Fortunately, Pat does have access to a family doctor and emergency department with a CT scanner all of which are helpful for managing symptoms (such as the provision of opioid medication) and providing urgent diagnosis in the event of sudden changes in Pat’s condition. However, Pat will need to travel access medical resources, including the cancer center for consultations prior to starting any treatment as well as for the treatment itself which will likely require extensive and repeated travel resulting in costs both financial, and in terms of time commitment. Therefore, Pat’s geographic distance from the cancer center can be described as a source of poor fit between Pat’s physical characteristics and the biomedical assets.

### Identifying Opportunities to Improve Fit

Beginning with the fit between Pat’s non-physical characteristics and the healthcare system, Pat’s experience may have been improved with additional information about lung cancer, treatments, diagnosis, and direction to credible and applicable internet resources (6-8). Additionally, referral specifically to psycho-social support at the cancer center at the time of referral may have provided Pat with additional and meaningful supports given Pat’s relative state of social isolation (8). In the same way, providing Pat with information about medical use of opioids, risks of addiction, and complementary approaches to opioid analgesia (such as with over-the-counter non-opioid analgesia to reduce opioid use) may have helped Pat feel more in control and positive and less ashamed and worried about taking opioid medications (9, 10). These interventions represent opportunities to improve fit between Pat’s non-physical characteristics, including the coping strategies Pat is using, and the assets available to Pat through the healthcare system.

From a systems perspective, this analysis of Pat’s case suggests that taking steps to identify who may benefit from additional psycho-social and informational supports at the time of cancer diagnosis before formal assessment at a cancer center is important. Each patient has a unique combination of informal caregiver supports, personal beliefs about cancer, and approaches to coping. Additionally, each patient interacts with a different combination of healthcare professionals during their cancer journey, whose ability and availability to identify and meet psycho-social issues and informational needs vary. While screening for psycho-social distress is increasingly becoming part of routine practice at many cancer centers (11), the mismatch between the non-biomedical needs of a patient and healthcare professionals’ ability to address these needs outside the context of a cancer center presents an opportunity to improve fit, especially early in the cancer journey prior to intake and assessment at a specialized cancer center.

In terms of physical characteristics, while the specific details of Pat’s lung cancer are not known aside from the fact that it can be considered stage IV, Pat’s geographic location will play a role in the cancer journey. Pat will likely be required to travel to the cancer center for treatment or for medical assessments on multiple occasions, sometimes with only a few days’ notice. In terms of bridging the distance to the cancer center, Pat will incur a number of costs both in terms of time, money, and possibly emotional stress (12). For visits at the cancer center Pat will have to travel both to and from the cancer center, navigate a possibly unfamiliar city, and find parking, meals, and even overnight lodgings. The role of provision of care using virtual or telehealth is certainly important for patients like Pat and balancing when in-person visits at the cancer center can be augmented with telephone or video-conferencing appointments is important (13). Additionally, ensuring that informational resources are available online, that patients are aware of them, and have assistance in overcoming barriers to access is especially important for those that have limited in-person access to a specialized cancer center (6).

## Case 2: Charlie

Charlie is 45 years old and lives with Sam and Kris. The three of them moved to a new city together in a different region of the country for Kris’s work a few months before Charlie was diagnosed with cancer where they didn’t know anyone. Sam is Charlie’s parent and has early dementia. Sam is safe to be alone for small periods of time but gets upset and confused if left alone for more than an hour. Charlie is the primary caregiver for Sam.

Charlie was diagnosed with colon cancer a few months ago and underwent surgery to remove the tumor and a number of lymph nodes. Charlie has received the third of eight injections of chemotherapy for colon cancer ten days ago. Each cycle of Charlie’s chemotherapy consists of injections every three weeks and pills that are taken twice a day for two weeks followed by one week off. Before the cancer diagnosis, Charlie worked at home doing freelance website design. This made it easy to look after Sam and earn an income. Charlie has continued to do freelance work during treatment on “good days”, albeit at a slower rate for accommodating clients. When Charlie was recovering from surgery Kris, Charlie’s spouse, took vacation days to stay home from work to help take care of both Sam and Charlie. The vacation days ran out before Charlie started chemotherapy and Kris had to go back to work as an electrician. Kris’s boss gave Kris permission to take half days when someone needed to be with Sam while Charlie went to doctors’ appointments and chemotherapy injections. Charlie’s first two cycles were difficult. Charlie had a lot of diarrhea, mouth sores, and abdominal pain. Charlies also experienced a lot of fatigue and struggled to get out of bed somedays. Charlie had to go to the ER for hydration in the middle of the night with the last cycle after nearly passing out after getting out of bed to fast.

Today Charlie is home alone with Sam. Charlie and Sam just ate lunch after spending the morning on the couch watching a movie. After finishing their lunch, Charlie suddenly starts to feel flushed, lightheaded, nauseas, and rushes to the bathroom to vomit. This hasn’t happened before. Charlie checks an oral temperature, and it’s 38.5 C. Charlie remembers being told to go to the ER immediately if an oral temperature is ever 38.3 C or over. Charlie calls Kris to see if Kris can come home and be with Sam because a trip to the ER could take many hours. Kris and Sam weren’t allowed to be with Charlie the last time they visited the emergency room due to the COVID-19 pandemic and Charlie isn’t sure if the rules have changed since then. Charlie knows it’s not safe to leave Sam alone. Kris is working at a rural job site with poor cell service and there is no answer. Charlie leaves a voicemail. Charlie checks another temperature. It’s 38.9 C.

Charlie calls the nurse that works in the clinic with the medical oncologist who prescribed the chemotherapy. There is no answer the phone goes straight to a voicemail message instructing the caller to “leave a message after the tone, and in the event of an emergency call 911 or proceed to the nearest emergency department”. Charlie leaves a voicemail asking what to do stating she can’t leave Sam alone at home to go to the emergency room. Charlie is panicking. Sam tells Charlie to go the emergency department and says, “I’ll be fine alone”. But Charlie knows that Kris might not be home for hours and that an emergency room visit could take until the early morning, if not longer.

Not knowing what else to do, Charlie calls, Jerry. Jerry and Charlie sometimes collaborate on website designs. They don’t have a personal relationship, but Jerry is one of the few people Charlie knows in the new city. Jerry doesn’t know that Charlie is being treated for cancer, that Sam lives with Charlie, or that Sam has dementia. Jerry doesn’t answer. Flustered, Charlie leaves a somewhat rambling voicemail message trying to explain the situation while holding back tears. Charlie ends the message by asking if Jerry can come look after Sam.

Charlie is starting to feel worse. It’s been about an hour since the nausea and fever started. Charlie has vomited again and is starting to feel shaky. Sam can tell Charlie isn’t feeling well and starts getting upset. Sam bursts out in tears “I’m ***your*** parent, I’m supposed to be looking after ***you***. I’m calling 911. Don’t worry about me. I’ll be fine”. Charlie tries to get up from the couch to stop Sam because Charlie is not leaving Sam alone – but gets dizzy and slumps back on the couch.

Ten minutes later the ambulance arrives. Charlie is too weak to get up from the couch and is not answering questions coherently. Sam tells the paramedic that Charlie is sick and shows them where Charlie’s pills are. As the paramedics load Charlie into the ambulance, Charlie’s phone vibrates on the kitchen table with a text from Jerry: “What’s your address? I’m on my way”.

### Deconstructing Charlie

*See Appendix C for the analysis of Charlie’s example presented using the Fit Theory Template.*

Charlie’s case highlights the challenge of caring for dependents while living with a personal diagnosis of cancer as well as the role of informal caregivers have in helping patients fulfill their various roles. Charlie has been told that a fever after receiving chemotherapy is a medical emergency and needs to be treated immediately. At the same time, Charlie is responsible for caring for an elderly parent who can’t be safely left alone and there are no additional supports available. At the start of the scenario, the fit between Charlie’s physical characteristics including symptoms of fever, nausea, vomiting, diarrhea, and the available biomedical assets is not necessary a problem if Charlie is able to access immediate medical attention. However, Charlie’s non-physical characteristic of being responsible for Sam’s wellbeing interacts with the ability of Charlie to access biomedical medical assets in a timely way. Because Charlie was unable to reach Kris or Jerry, Sam will be potentially left alone for several hours which could be dangerous. Additionally, Charlie was not able to get to medical attention before becoming very ill which could ultimately result in significant morbidity and even mortality for Charlie.

### Identifying Opportunities to Improve Fit

This example highlights an instance of poor fit related to an interaction between non-physical characteristics, physical characteristics, and biomedical assets. Charlie’s physical characteristics in terms of the new evolving symptoms including fever in the setting of recent chemotherapy indicate that Charlie needs urgent medical attention (14). But Charlie’s responsibility to Sam is a non-physical characteristic that limits Charlie’s ability to access medical attention in this case. Additionally, the nature of ER visits, including wait times, and, in the context of the COVID19 pandemic, potential limitations on whether family members can accompany a patient in the ER, make accessing the ER a relatively poor fit.

If it weren’t for Charlie’s responsibility to Sam, Charlie’s journey to the ER would have been more straightforward and without delay. While Kris would have been able to take care of Sam, Kris wasn’t able to be reached which was likely unexpected. Ideally, Charlie, Sam and Kris would have developed an emergency plan about how to care for Sam if Charlie needed to get emergency help and Kris wasn’t available. This could have involved asking Jerry ahead of time for assistance in case of emergency and giving Jerry the address for Charlie’s house. On a systems level, this case examples suggests a role for identifying patients with dependents and developing plans to care for the dependents in the event of medical emergency, emergency plans is a strategy that could be developed and incorporated into cancer care appointments for patient’s receiving new treatments.

# Concluding Notes on Applying Fit Theory

The above cases provide an illustration of how Fit Theory can be applied to case examples to better understand the issues faced by patients that define much of the cancer experience. Importantly, Fit Theory provides a framework for guiding exploration of the many challenges faced by cancer patients, their families, and for facilitating the identification opportunities for improving fit between the unique characteristics of patients and available health services on a health systems level.

Notably, the analysis of the narratives and the identification opportunities for improving fit that result are expected to differ depending on who is conducting the analysis. It is assumed that interdisciplinary differences will exist in terms of how the narratives are analyzed and what characteristics, assets, and sources of good/poor fit are identified. Additionally, what is available in the geo-graphic region where the examples are being discussed may provide additional context. For instance, when examined through the lens of what is available in some rural communities, the challenges faced by Pat may be interpreted differently than in other communities leading to the identification of different opportunities for improving fit. But this underscores the potential power of Fit Theory as theory that can be applied in the real world. While it is grounded in the lived experience of cancer patients in a specific context (i.e., Manitoba, Canada during the COVID-19 pandemic), it is general enough to be applied in other cancer contexts, and provides the applier with control over how to best to use the concepts in their own context (4).

While the work to generate Fit Theory was conducted with the intention of informing health services delivery, it is likely that it may be useful in the educational setting. Instructors are encouraged to consider how the case examples provided here may be incorporated into small group learning sessions. Learning activities might include working with learners to develop their own analysis of the cases provided, including the identification of opportunities for improving fit. Additionally, educators are encouraged to create their own narrative examples for analysis, using either entirely real-world cases, cases comprised of a composite of real-world events (such as the two presented here), or fictional cases developed to demonstrate a learning point and facilitate group discussion.

**References**

1. Birks M, Mills J. Application of Grounded Theory. In: Glaser B, editor. Applying Grounded Theory: A Neglected Option. 1st ed. Mill Valley, California: Sociology Press; 2014.

2. Glaser B. Professional Applying of Grounded Theory. In: Glaser B, editor. Appying Grounded Theory: A Neglected Option. 1st ed. Mill Valley, California: Sociology Press; 2014.

3. Brown B. Shame Resilience Theory: A Grounded Theory Study on Women and Shame. Families in Society. 2006;87(1):43-52. doi:10.1606/1044-3894.3483.

4. Glaser B. Applying Grounded Theory. 1st ed. Mill Valley, California: Sociology Press; 2014.

5. Thiessen M, Hack TF, Pitz M, Anderson M. A model of identity grounded in the acute season of survivorship. Psychooncology. 2018;27(10):2412-8. doi:10.1002/pon.4842.

6. Thiessen M, Sinclair S, Tang PA, Raffin Bouchal S. Information Access and Use by Patients With Cancer and Their Friends and Family: Development of a Grounded Theory. J Med Internet Res. 2020;22(10):e20510. doi:10.2196/20510.

7. Germeni E, Schulz PJ. Information seeking and avoidance throughout the cancer patient journey: two sides of the same coin? A synthesis of qualitative studies. Psychooncology. 2014;23(12):1373-81. doi:10.1002/pon.3575.

8. Gao Y, Zhu L, Xie J, Liu A, Ding Y, Yao J. Unmet needs from the first diagnosis of cancer until the end of medical treatment: A longitudinal study. Psychooncology. 2021;30(4):554-63. doi:10.1002/pon.5602.

9. Graczyk M, Borkowska A, Krajnik M. Why patients are afraid of opioid analgesics: a study on opioid perception in patients with chronic pain. Pol Arch Intern Med. 2018;128(2):89-97. doi:10.20452/pamw.4167.

10. Bulls HW, Chu E, Goodin BR, Liebschutz JM, Wozniak A, Schenker Y, et al. Framework for opioid stigma in cancer pain. Pain. 2022;163(2):e182-e9. doi:10.1097/j.pain.0000000000002343.

11. Salmon P, Clark L, McGrath E, Fisher P. Screening for psychological distress in cancer: renewing the research agenda. Psychooncology. 2015;24(3):262-8. doi:10.1002/pon.3640.

12. Rocque GB, Williams CP, Miller HD, Azuero A, Wheeler SB, Pisu M, et al. Impact of Travel Time on Health Care Costs and Resource Use by Phase of Care for Older Patients With Cancer. J Clin Oncol. 2019;37(22):1935-45. doi:10.1200/JCO.19.00175.

13. Singh S, Fletcher GG, Yao X, Sussman J. Virtual Care in Patients with Cancer: A Systematic Review. Curr Oncol. 2021;28(5):3488-506. doi:10.3390/curroncol28050301.

14. Zimmer AJ, Freifeld AG. Optimal Management of Neutropenic Fever in Patients With Cancer. J Oncol Pract. 2019;15(1):19-24. doi:10.1200/JOP.18.00269.

# Appendix A: Fit Theory Analysis Framework Blank Template

**Consequences:**

| **Human Assets** |  | **Non-Physical Characteristics** |
| --- | --- | --- |
|  |  |  |
| **Biomedical Assets** |  | **Physical Characteristics** |

| **Informal Caregiver Assets** |
| --- |

**Impact on Fit**

Source of Good Fit

Source of Poor Fit

Unknown

# Appendix B: “Pat” Fit Theory Analysis Example

**Consequences:** Social isolation, hyper-vigilance (re: missing appointment notification), anxiety and fear about prognosis and treatment, hopeful about immunotherapy, increased travel costs and time for assessments/treatments at the cancer center.

| **Human Assets**  Family physician’s bedside manner  Family physician’s knowledge about cancer, pain medication, and ability to share it |  | **Non-Physical Characteristics**  Recent loss of partner to cancer  Knowledge about lung cancer  Beliefs about opioid medications  Coping skills |
| --- | --- | --- |
|  |  |  |
| **Biomedical Assets**  Local ER/Family doctor  Opioid Medications  Cancer Center Location  Treatments Available  Formal Cancer Informational Resource Infrastructure |  | **Physical Characteristics**  Age (50 years old)  Lung Cancer Diagnosis Details (stage IV, type)  Symptoms: Pain, weight loss, hemoptysis  Location: rural community, long distance from cancer center |

| **Informal Caregiver Assets**  Unclear what informal caregiver assets are available, as Pat has kept the diagnosis a secret. |
| --- |

**Impact on Fit**

Source of Good Fit

Source of Poor Fit

Unknown

# Appendix C: “Charlie” Fit Theory Analysis Example

**Consequences:** Anxiety, fear, increased symptoms and worsened biomedical outcomes

| **Human Assets**  (Not Evident in Narrative) |  | **Non-Physical Characteristics**  Caregiver for elderly parent with dementia  Flexible job |
| --- | --- | --- |
|  |  |  |
| **Biomedical Assets**  Emergency Department  Chemotherapy |  | **Physical Characteristics**  Age (45 years old)  Colon cancer, curative intent treatment  Symptoms: Nausea, vomiting, fever  Last chemotherapy ten days ago (risk of febrile neutropenia) |

| **Informal Caregiver Assets**  Supportive Spouse with supportive employer  but currently not accessible |
| --- |

**Impact on Fit**

Source of Good Fit

Source of Poor Fit

Unknown
